# Lymphoepithelioma-like carcinoma of the urinary bladder: report of a rare case

**DOI:** 10.4103/0256-4947.57173

**Published:** 2009

**Authors:** Naorem G. Singh, Abul Ala Syed R. Mannan, Amre A. Rifaat, Mirza Kahvic

**Affiliations:** From the Department of Pathology, Al-Jahra Hospital, Jahra, Kuwait

## Abstract

Lymphoepithelioma-like carcinoma (LELC) is a rare malignant neoplasm in the urinary bladder, which can histologically mimic lymphoma, poorly differentiated invasive transitional cell carcinoma or poorly differentiated squamous cell carcinoma with a lymphoplasmacytic background. A urinary bladder tumor was identified in a 65-year-old man suffering from hematuria for several weeks. Transurethral biopsy revealed an undifferentiated tumor with prominent lymphocytes and plasma cell infiltration. Immunohistochemical evaluation showed positive staining for cytokeratin and epithelial membrane antigen. Subsequent radical cystectomy showed pure LELC. We present the case to highlight the significance of recognizing this unusual bladder tumor and discuss the important differential diagnosis, treatment options and prognosis.

Lymphoepithelioma is the term used for describing an undifferentiated carcinoma of the nasopharynx, histologically characterized by the presence of prominent lymphoid aggregates in the background. Carcinomas with similar histologic features arising outside the nasopharynx are called lymphoepithelioma-like carcinoma (LELC), which are reported in various organs such as the thymus, salivary gland, and cervix. Involvement of the urinary bladder, first reported by Zuckerberg et al in 1991,^1^ is uncommon, with a reported incidence between 0.4% and 1.3% of all bladder carcinomas.^2^ The tumor can occur either in pure form or in association With conventional urothelial carcinoma.^3^ The pure form is considered to have a better prognosis and exhibits a good response to chemotherapy.^2^–^5^ It is important to differentiate LELC from non-Hodgkin lymphoma, poorly differentiated invasive transitional cell carcinoma and poorly differentiated squamous cell carcinoma with lymphoplasmacytic infiltrate in the background. We report a case of pure LELC of the urinary bladder in a 65-year-old man and discuss the important differential diagnosis, various treatment options and the prognosis of this unusual tumor. To our knowledge, this is the first reported case of LELC of the urinary bladder from the Middle East.

## CASE

**Figure 1:**
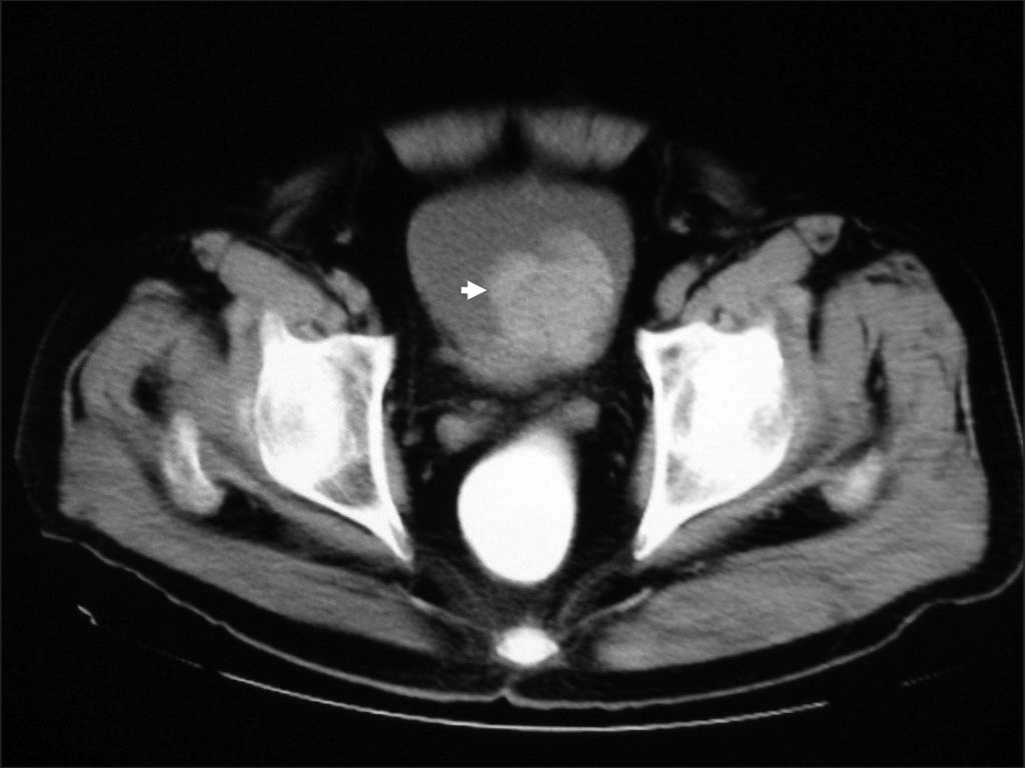
Contrast-enhanced CT scan showing moderately enhancing lobular intravesical mass lesion (arrow) arising from the base of the urinary bladder, involving the right vesicoureteric junction.

**Figure 2:**
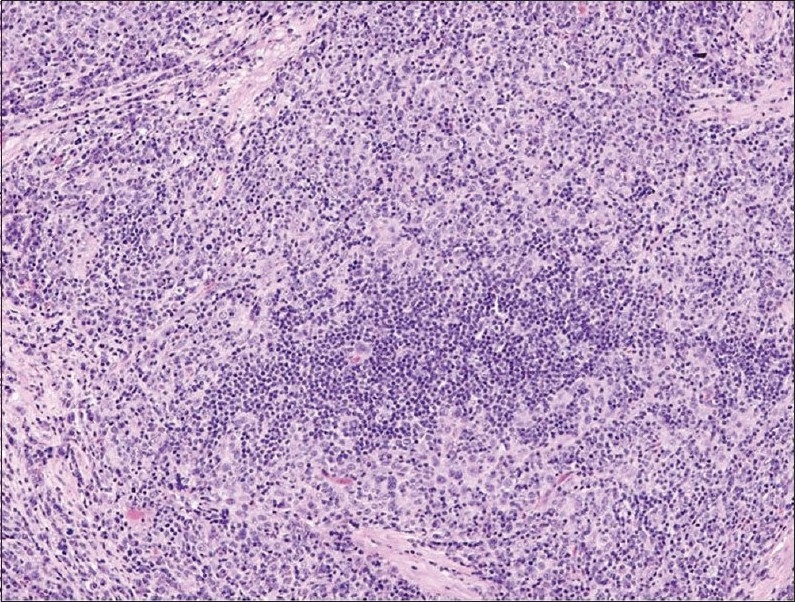
Photomicrograph demonstrating diffuse sheets of pleomorphic tumor cells admixed with prominent inflammatory infiltrate (hematoxylin-eosin stain ×200).

**Figure 3:**
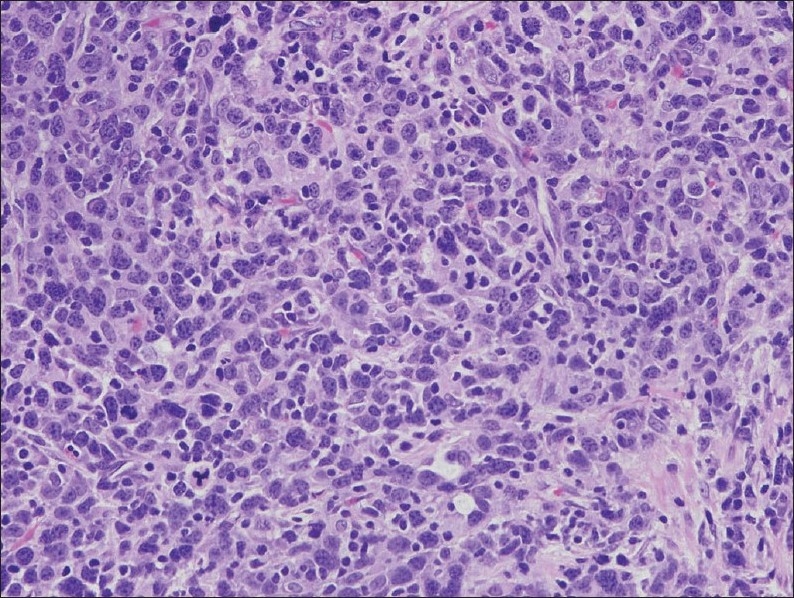
Undifferentiated tumor cells with vesicular nuclei and indistinct cell margin along with few interspersed lymphocytes and plasma cells (hematoxylin-eosin stain ×400).

**Figure 4:**
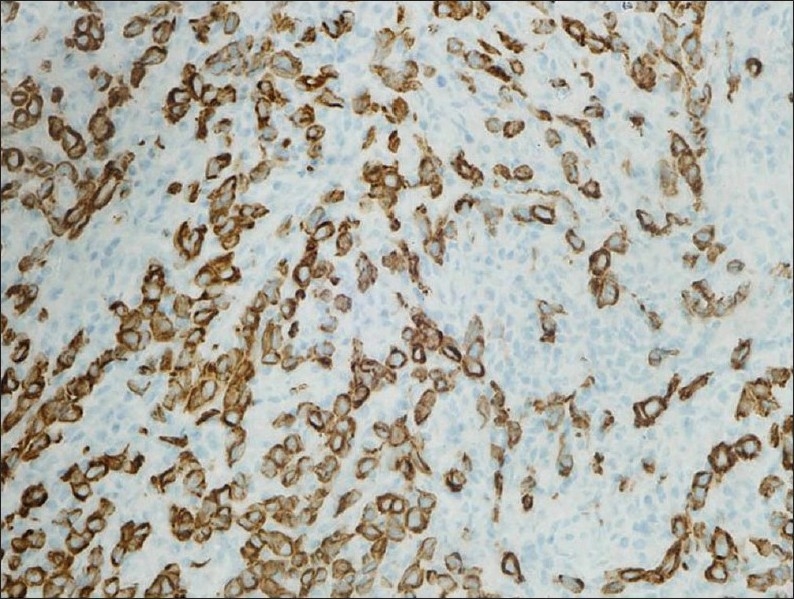
Immunohistochemical stain for pancytokeratin, highlighting tumor cells with unstained lymphocytes in the background (immunoperoxidase stain ×400).

A 65-year-old man presented with a history of recurrent painless hematuria for several weeks. There was no history of other urological complaints. Cystoscopy showed a sessile ulcerated lesion measuring about 3 cm in diameter, in the right posterolateral wall of the bladder. A CT scan revealed a soft tissue tumor mass in the right posterolateral aspect of the urinary bladder wall involving the right ureteric orifice, with a consequent right-sided hydroureter (Figure 1). There was no evidence of perivesical spread of the tumor or metastatic disease elsewhere. Transurethral resection of the tumor was attempted. The biopsy was reported as LELC with infiltration into the muscularis propria. Subsequently, a radical cystectomy obtained a specimen that measured 8.5×7×4 cm. A cut section revealed an ulcerated tumor mass measuring 3×3×1.5 cm at the right posterolateral wall of the bladder. On microscopy, the tumor was composed of diffuse sheets and cords of undifferentiated cells having large pleomorphic nuclei with coarse chromatin (Figures 2 and 3). Many of the nuclei had prominent nucleoli. The cytoplasm was moderate with poorly defined borders. The background showed dense lymphoplasmacytic infiltration with occasional eosinophils. The tumor showed transmural infiltration, but not invading the perivesical adipose tissue. There was no evidence of an in situ component in any of the examined sections. The entire tumor demonstrated similar morphology, without any associated component of conventional urothelial type carcinoma, or any other subtypes, such as squamous cell carcinoma or adenocarcinoma. The tumor cells were positive for pan cytokeratin (CK) (Figure 4), CK7, CK 8 and Epithelial Membrane Antigen, but negative for chromogranin, vimentin and desmin. A diagnosis of pure LELC with evidence of muscular invasion was entertained. The patient had an uneventful post operative period. He is currently on follow-up and is free of symptoms, 12 months after the surgery.

## DISCUSSION

LELC is a rare variant of infiltrating urothelial carcinoma, first described by Zuckerberg et al in 1991.^1^ Since then there have been only a few relatively small studies on LELC of urinary bladder.^2^–^8^ The largest study was reported by Tamas et al^8^ who described 17 pure 13 mixed LELC. Prior to that Lopez-Beltran et al^5^ reported 3 pure, 6 predominant and 4 focal LELCs of the urinary bladder. Amin et al^3^ reported 3 pure, 5 predominant and 3 focal LELCs and Holmang et al^6^ described 3 pure, 5 predominant and 3 focal cases of LELC. To our knowledge, LELC of the urinary bladder has not been reported from the Middle East to date.

The exact pathogenesis of this tumor is not well established. Epstein-Barr virus (EBV) is frequently associated with lymphoepithelioma of the nasopharynx and LELC arising in the lung, stomach, thymus and salivary gland.^1^,^2^,^8^,^9^ However, such an association has not been documented for LELC of the urinary bladder. Hybridization with EBV-encoded RNA (EBER) has been reported to be negative.^5^,^8^,^9^ It has been suggested that abnormalities of p53 regulation might be crucial in the pathogenesis of LELC of the urinary bladder.^10^ Though the exact origin of these carcinomas is not well known, the expression of common urothelial markers suggest that they are probably modified urothelial cells that have derived from stem cells.^5^,^6^

The tumor usually presents in late adulthood. Most patients present with painless hematuria and are stage T2-T3 at diagnosis.^5^,^6^,^8^ Histological features of LELC closely resemble lymphoepithelioma of the nasopharynx, with the tumor growing in nests, sheets or cords of large undifferentiated malignant epithelial cells within a dense inflammatory background, comprising mature lymphocytes, plasma cells, and rarely neutrophils and eosinophils. The background lymphocytes are usually an admixture of B and T lymphocytes.^11^ Amin et al^3^ categorized the LELC of bladder into three subgroups as pure, predominant (more than 50% lymphoepithelial component) and focal (less than 50% lymphoepithelial component). Invasive urothelial carcinoma usually accompanies predominant or focal LELC.^3^,^5^,^8^ Though uncommon, the tumor may have accompanying adenocarcinoma and squamous cell carcinoma as well.^5^,^8^ The overlying or adjacent urothelium may show urothelial dysplasia or carcinoma in situ.^2^,^8^ The present case was a pure LELC. A correct diagnosis on a urine cytology specimen can be particularly challenging, considering the rarity of the tumor. Cai and Parwani^12^ reported cytologic findings in two cases of LELC of the bladder, which were confirmed by histopathological examination of the resected tumor. Useful cytomorphologic features include the presence of large tumor cells with a high nuclear-to-cytoplasmic ratio, vesicular chromatin and prominent nucleoli, presenting as single cells or intermixed with inflammatory cells.^12^

Pure LELC must be distinguished from reactive inflammatory lesions or lymphoma. Primary lymphoma of the bladder is extremely rare, and has an entirely different therapeutic approach.^3^ Immunohistochemistry for cytokeratin and lymphoid markers can help in resolving this differential diagnosis. Owing to the presence of a prominent inflammatory background, the neoplastic cells may be assumed to be reactive histiocytes and the lesion may be misdiagnosed as chronic cystitis.^1^,^3^ Hence, a dense lymphoid infiltrate in a urinary bladder biopsy should alert the pathologist to closely look for neoplastic cells. Immunohistochemistry for epithelial markers can be helpful in highlighting the neoplastic cells in such instances. Other important differential diagnoses include poorly differentiated invasive transitional cell carcinoma or poorly differentiated squamous cell carcinoma with associated dense lymphoplasmacytic infiltrate.^3^,^13^ Sometimes, it might be problematic to differentiate LELC from small cell carcinoma of the urinary bladder or prostate in small, improperly fixed biopsy specimens with crush artifacts.^1^,^3^

Most of the previous studies have suggested that LELC has a relatively favorable prognosis when in pure or predominant forms with reported rates of metastasis ranging from only 12% to 15%.^2^,^3^,^5^ Amin et al^3^ reported on three patients having pure LELC and five patients having predominant LELC, all of whom showed no evidence of disease (2-72 months). In contrast, the two patients with focal LELC succumbed to metastatic disease 6 and 84 months after diagnosis. Similarly all the three patients in the study by Holmang et al^6^ with focal LELC died of the disease (9-68 months), compared to none of the six patients with pure or predominant LELC (13 months to 18 years). In the study by Lopez-Beltran et al,^5^ all three patients with pure and 4/6 patients with predominant LELC were alive, while all four patients having focal LELC died of disease. However, Tamas et al^8^ did not find any difference in prognosis between pure and mixed LELC. Their study demonstrated that LELC treated by cystectomy has a similar prognosis to ordinary urothelial carcinoma and does not differ between pure and mixed cases. In cases with cystectomy, the overall 5-year actuarial recurrence-free risk was 59% (62% and 57%, for pure and mixed LELC, respectively). The rationale behind this apparent better prognosis, however, has not been well investigated. It is known that tumors with lymphoid infiltration have a comparatively better prognosis than those without it. Intense immune response generated by these lymphoid cells against the tumor may play an important role in this regard.^3^

The paucity of published literature suggests that there is limited experience in therapeutic approaches to LELC of the bladder. The pure/predominant form may respond to chemotherapy.^3^–^5^,^8^ In the study by Amin et al,^3^ four of the pure/predominant LELC were treated with transurethral resection and chemotherapy and all showed no evidence of disease. Dinney et al^4^ has shown a complete response to chemotherapy and transurethral resection of the bladder tumor in 3 cases of muscle invasive LELC. In another study by Lopez-Beltran et al^5^ 2 of their 13 patients received chemotherapy and both showed no evidence of disease at 21 and 47 months. In a recent study by Tamas et al,^8^ of the three pure cases treated by chemotherapy, two were free of disease at 4 and 65 months and the third had recurrent disease at 17 months. Our case underwent radical cystectomy without chemotherapy and was free of disease 12 months after surgery.

In conclusion, LELC of the bladder is rare and should be ruled out in bladder biopsies that show dense lymphoid infiltrate. The differential diagnosis is usually lymphoma, poorly differentiated invasive transitional cell carcinoma and poorly differentiated squamous cell carcinoma with lymphoplasmacytic background. Existing data suggest the pure form responds well to chemotherapy and has a better prognosis.
